# Impact of statin related media coverage on use of statins: interrupted time series analysis with UK primary care data

**DOI:** 10.1136/bmj.i3283

**Published:** 2016-06-28

**Authors:** Anthony Matthews, Emily Herrett, Antonio Gasparrini, Tjeerd Van Staa, Ben Goldacre, Liam Smeeth, Krishnan Bhaskaran

**Affiliations:** 1 Department of Non-Communicable Diseases Epidemiology, London School of Hygiene and Tropical Medicine, London, UK; 2 Department of Social and Environmental Health Research, London School of Hygiene and Tropical Medicine, London, UK; 3 Health eResearch Centre, Farr Institute for Health Informatics Research, University of Manchester, Manchester, UK; 4 Division of Pharmacoepidemiology and Clinical Pharmacology, Utrecht Institute of Pharmaceutical Sciences, Utrect, Netherlands

## Abstract

**Objective** To quantify how a period of intense media coverage of controversy over the risk:benefit balance of statins affected their use.

**Design** Interrupted time series analysis of prospectively collected electronic data from primary care.

**Setting** Clinical Practice Research Datalink (CPRD) in the United Kingdom.

**Participants** Patients newly eligible for or currently taking statins for primary and secondary cardiovascular disease prevention in each month in January 2011-March 2015.

**Main outcome measures** Adjusted odds ratios for starting/stopping taking statins after the media coverage (October 2013-March 2014).

**Results** There was no evidence that the period of high media coverage was associated with changes in statin initiation among patients with a high recorded risk score for cardiovascular disease (primary prevention) or a recent cardiovascular event (secondary prevention) (odds ratio 0.99 (95% confidence interval 0.87 to 1.13; P=0.92) and 1.04 (0.92 to 1.18; P=0.54), respectively), though there was a decrease in the overall proportion of patients with a recorded risk score. Patients already taking statins were more likely to stop taking them for both primary and secondary prevention after the high media coverage period (1.11 (1.05 to 1.18; P<0.001) and 1.12 (1.04 to 1.21; P=0.003), respectively). Stratified analyses showed that older patients and those with a longer continuous prescription were more likely to stop taking statins after the media coverage. In post hoc analyses, the increased rates of cessation were no longer observed after six months.

**Conclusions** A period of intense public discussion over the risks:benefit balance of statins, covered widely in the media, was followed by a transient rise in the proportion of people who stopped taking statins. This research highlights the potential for widely covered health stories in the lay media to impact on healthcare related behaviour.

## Introduction

Statins reduce the risk of cardiovascular disease^1^ and are widely recommended as part of the strategy for primary and secondary prevention.^2^ ^3^ ^4^ ^5^ ^6^ Severe adverse effects associated with statins are extremely rare,^7^ but concerns over purportedly high rates of side effects such as muscle pain and weakness have been raised in the academic press and reported in the national media. In October 2013, two articles published in *The BMJ* were perceived as critical of statins, with one suggesting that side effects might outweigh the overall health benefits in patients at low and intermediate risk.^8^ ^9^ Although the comments on rates of side effects were based on evidence from non-blinded observational data, and the articles were focused on the benefit:risk ratio in those with low risk, they generated extensive and broader discussion in the media about statins. The debate peaked in March 2014, when most national media outlets in the United Kingdom covered the subject.^10^ ^11^ Media coverage of these articles was probably intensified because of the impending changes in the guidelines proposed by the UK National Institute for Health and Care Excellence (NICE, July 2014), which broadened eligibility for statins from patients with a high (≥20%) 10 year risk of cardiovascular disease to those with intermediate (≥10%) 10 year risk.

As a society we are increasingly exposed to numerous and disparate sources of health information, and it has been shown that this bombardment leads to a lack of clarity about which sources patients and others should trust.^12^ Studies in Denmark, Australia, Turkey, and France have suggested that media debate about side effects of statins has led to measurable effects on certain aspects of use,^13^ ^14^ ^15^ ^16^ and qualitative work has shown that concerns over side effects and a desire for clearer information regarding the risks and benefits can affect use in patients in the UK.^17^ ^18^ ^19^ No large studies to date, however, have comprehensively evaluated the effects of media debates about treatment with statins on prescribing for both the primary and secondary prevention of cardiovascular disease and the consequences for public health.

Using prescribing data from routinely collected UK primary care records, we quantified the potential association between the debate in the media about the side effects of statins and initiation and cessation of treatment in UK primary care for both primary and secondary prevention of cardiovascular disease. We investigated whether any potential media effects differed by key patient level characteristics and estimated the public health impact of any changes in patterns of use that might have arisen from the controversy, in particular the resulting number of excess cardiovascular events.

## Methods

### Study design and setting

This ecological interrupted time series study used prospectively collected data from the UK Clinical Practice Research Datalink (CPRD), a primary care database containing anonymised data from about 6.9% of the UK population.^20^ ^21^ General practitioners play a key role in the UK healthcare system as they are responsible for primary healthcare and specialist referrals. The CPRD includes prescriptions and clinical diagnoses from primary care, as well as diagnoses from secondary care that are typically fed back to general practitioners. Those represented in the database are broadly representative of the UK population in terms of age and sex.^21^ In the interrupted time series design, population level outcomes (in this case, proportions of people starting/stopping statins) are calculated over time, and then statistical regression techniques are used to investigate how trends in these outcomes are affected by a population level exposure that occurs in a single well defined time period (here, widespread media coverage about statins over a six month period)—that is, the exposure is viewed as a potential “interruption” to the underlying trends in the outcome(s) over time.^22^

We produced a code list for statins by identifying all drugs that included the word “statin” in either the product name or the drug substance name. The proportions of patients initiating and stopping statins were calculated for each month from January 2011 to March 2015. Definitions for our study populations and ascertainment of statin initiation and cessation are described below.

### Statin initiation

#### Study population

For each calendar month, we identified all individuals aged >40 registered at their general practice for at least a year, with no previous recorded prescriptions for a statin, and no previous cardiovascular disease events, and who had either a newly recorded 10 year cardiovascular risk score (hereafter referred to as simply 10 year risk score) of >20% (appendix part 1) (that is, eligible to start taking a statin for primary prevention of cardiovascular disease) or an incident cardiovascular event (that is, eligible to start taking a statin for secondary prevention). Incident events were defined as a first record of coronary heart disease (myocardial infarction, angina, revascularisation procedures), cerebrovascular disease (stroke, transient ischaemic attack), or peripheral vascular disease (abdominal aortic aneurism and intermittent claudication) dated at least a year after the start of a patient’s CPRD follow-up. These events were identified within patient records by searching for NHS Read codes corresponding to any of these diagnoses; we used code lists developed for the CALIBER programme.^23^

#### Defining initiation

We then calculated the proportion of patients who started taking statins for primary and secondary prevention separately for each calendar month throughout the study period. Patients were defined as starting a statin for primary prevention if they had received a first prescription within 28 days of the date of the risk score being recorded. The denominator was all patients eligible to start treatment for primary prevention (as above) who remained alive, under follow-up, and free from cardiovascular disease for the full 28 day period after their risk score. Patients were defined as starting a statin for secondary prevention if they received a first prescription in primary care ≤60 days after their first cardiovascular event; the 60 day grace period was chosen to allow for a period of admission to hospital, based on preliminary analyses (appendix part 2). The denominator for this calculation was all those eligible to start treatment for secondary prevention (as above) who remained alive and under follow-up for the full 60 day grace period.

### Statin cessation

#### Study population

For each calendar month, we identified all individuals aged >40 and in receipt of a statin prescription that ended within that calendar month. Prescription end dates were calculated based on the date of prescription and quantity of tablets prescribed (appendix part 3). Patients could be included in more than one monthly cohort if they had multiple prescriptions ending during the study period. The study population was then stratified into those taking statins for primary prevention (defined as those with no record of a previous cardiovascular event) and those taking statins for secondary prevention (those with any previous event).

#### Defining cessation

We calculated the proportion of patients who stopped their statins each month: stopping was defined as receiving no further prescription within 28 days of the end date of the previous prescription. This 28 day grace period allowed time for patients with previously overlapping prescriptions to use their excess tablets, based on a preliminary analysis in which we identified all prescriptions from January 2011 to October 2013 and calculated that 90% of prescriptions were followed up with a new prescription within 28 days of the initial prescription ending. Only those remaining alive, under follow-up, and free from cardiovascular disease (for the primary prevention analysis) for the full 28 day grace period were included in the denominator.

### Period of exposure to high media coverage

We defined an exposure period of October 2013 to March 2014, and we compared patterns of statin initiation and cessation before and after this time period. The start date of the exposure was chosen to coincide with the publication of *The BMJ* papers about statins.^8^ ^9^ The end date was determined by carrying out a Google trend analytics search for the term “statin side effects” in the UK, which tracks the popularity of this search term over time, and by taking the date of peak searching for this term after October 2013 as the end of the exposure period (appendix part 4). This led to the choice of March 2014, which coincided with a spell of widespread coverage of the debate over statin side effects across most major national media outlets in the UK.

### Statistical analysis

We carried out an interrupted time series analysis using a generalised linear model with a binomial error structure, which accounts for the month by month variation in denominators (that is, the number of people eligible to initiate or stop taking a statin each month).^22^ Seasonal effects were accounted for by adjustment for calendar month,^24^ and first order lagged residuals were included to account for autocorrelation.^25^ Standard errors were scaled to account for overdispersion.^26^ Time was divided into three segments: before, during, and after the exposure period of high media coverage.

Within this modelling framework, we conducted separate analyses to investigate changes in statin initiation for primary prevention; statin initiation for secondary prevention; statin cessation in primary prevention; and statin cessation in secondary prevention. In each analysis, we looked at whether there was a step change in the log odds of initiating or stopping a statin after the exposure period compared with before, assuming an underlying linear month on month trend throughout the study period. We also investigated whether the underlying trend over time in the log odds of initiating/stopping a statin changed after the exposure period, compared with before. A Wald test was used to compare the trends (log odds ratio per month) before and after exposure. Linear predictions of the log odds and 95% confidence intervals of an event were calculated from the models and converted into probabilities, which we plotted along with a scatter of the raw proportion of patients that initiated/stopped treatment with a statin. We did not directly estimate trends during the exposure period itself because in this period individual level exposure to the coverage would probably have been dynamically changing, and the small number of data points in this period would have led to imprecise and inconclusive estimates.

We then investigated effect modification by the following prespecified factors: age group (40-49, 50-59, 60-69, 70-79, ≥80), sex, diabetes (identified with Read codes), and length of previous continuous prescription (up to six months, six months to one year, one to two years, two to four years, longer than four years). When stratifying by length of previous continuous prescription, we restricted the analysis to patients whose most recent continuous prescription began at least a year after their current registration into CPRD, to ensure that the initiation date was accurate and duration could be reliably categorised. By re-fitting the models with the monthly counts stratified by the above factors, and an interaction term added between the potential effect modifier and the parameter representing the post-exposure change in cessation, we examined each potential effect modifier in a separate model and generated P values with likelihood ratio tests. For ordinal covariates, we further explored effect modification by testing for linear trend.

### Post hoc analyses

After observing an increase in statin cessation that seemed to be transient in our primary analyses, we then separated post-exposure time into two periods of six months, and tested whether there was a difference between the modelled levels of cessation in each post-exposure period versus the pre-exposure period. We also carried out analyses investigating both a step change and trend change simultaneously after the exposure period, which allowed us to determine the monthly rate at which cessation fell after the initial level increase.

To examine changes in the application of risk scoring by GPs over time, we calculated the monthly proportion of patients in the whole of CPRD with any recorded 10 year risk score for cardiovascular disease, as well as the proportions of patients with very high (≥30%), high (20-30%), intermediate (10-20%), or low (<10%) risk scores recorded; these data were then analysed with similar methods as for the main analysis.

### Negative control analysis

In a preplanned negative control analysis, we replicated the main analyses using drugs prescribed for glaucoma (appendix part 5). Like statins, these drugs are given to those at high risk of disease as a preventive measure, are prescribed with similar frequency, and are typically intended to be continued for life after initiation. The purpose of this analysis was to ensure that any changes in statin use after the exposure period were not explained by broader unrelated underlying trends in prescriptions coincident with our exposure period or by other biases arising from the methods. In a second negative control analysis, we re-ran our main analyses using an alternative exposure period of 12 months earlier as we had no reason to expect any changes in prescribing trends around this time.

### Public health impact

To estimate the potential public health impact of changes in statin cessation, we compared the modelled cessation level in the first six months after the high media coverage exposure period (see “post hoc analyses” above) with the expected cessation level in the same period had there been no changes after exposure (that is, simply projecting the modelled “before” trend line for cessation forward). This estimated the number of patients who might have stopped taking a statin because of the controversy reported in the media, under the assumption of causality. We then estimated the number of excess cardiovascular events among these patients, assuming an average 10 year risk of 20% among those stopping, and assuming that statins would reduce risk by 19%, based on statin efficacy estimates from the Cholesterol Treatment Trialists’ Collaboration.^27^ We also used historical data from CPRD to estimate and take account of the proportion of patients who would have stopped taking statins or died in the following 10 years regardless of the media controversy. On the basis of results from a published study,^28^ we assumed that 66% of patients who stopped statins would restart within the following 12 months with no loss of protection. In a second calculation, to obtain an upper bound on the impact, we made the more pessimistic assumption that all those who stopped taking statins did not ever take them again. Full details of these calculations are given in appendix part 6.

All data analyses were carried out in Stata version 14, and all code lists are available at https://clinicalcodes.rss.mhs.man.ac.uk/medcodes/article/46/.

### Patient involvement

No patients were involved in setting the research question or the outcome measures, nor were they involved in developing plans for design or implementation of the study. No patients were asked to advise on interpretation or writing up of results. There are no plans to disseminate the results of the research to study participants or the relevant patient community

## Results

Throughout the study period we identified 88 010 records of a 10 year risk score of ≥20% for cardiovascular disease, 28 593 incident cardiovascular events, 9 286 148 prescriptions of statins for primary prevention, and 5 130 148 prescriptions of statins for secondary prevention. Table 1 provides an overview of study populations.

**Table 1 tbl1:** Characteristics of study populations from CPRD according initiation or cessation of statins for primary and secondary prevention of cardiovascular disease. Figures are numbers (percentage) unless stated otherwise

	Study populations			
	Primary initiation	Secondary initiation	Primary cessation	Secondary cessation
No of events*	88 010	28 593	9 286 148	5 130 148
No of outcomes†	20 249	17 207	751 243	328 595
No of patients	70 409	28 593	457 073	230 610
Men	48 136 (68.4)	16 512 (57.8)	237 802 (52.0)	137 776 (59.7)
Women	22 237 (31.6)	12 081 (42.2)	218 271 (48.0)	92 833 (40.3)
Indeterminate	0 (0)	0 (0)	1 (0)	1 (0)
Age (years)‡:				
40-49	2502 (3.6)	2396 (8.4)	39 806 (8.7)	7534 (3.3)
50-59	12 537 (17.9)	5377 (18.8)	95 485 (20.9)	26 533 (11.5)
60-69	30 491 (43.3)	7152 (25.0)	153 395 (33.6)	57 405 (24.9)
70-79	21 939 (31.2)	6306 (22.1)	116 008 (25.4)	73 013 (31.7)
≥80	2904 (4.1)	27 362 (5.8)	52 379 (11.5)	66 125 (28.7)
Median (IQR)	66 (61-72)	69 (58-80)	66 (58-74)	73 (64-81)
Diabetes§	5644 (8.0)	2466 (8.62)	114 910 (31.7)	63 868 (27.7)

IQR=interquartile range.

*No of opportunities for patients to either initiate or stop statins, either risk score >20% or incident cardiovascular event in initiation populations, or end of statin prescription in cessation populations.

†No of occurrences of initiation in initiation populations and No of occurrences of stopping in cessation populations.

‡Age at first risk score >20% or incident cardiovascular event in initiation populations and age at first prescription in study period in cessation populations.

§Diagnosis of diabetes before or within study period.

Figure 1 shows the estimated step change in the likelihood of patients initiating and stopping taking a statin for primary and secondary prevention after the exposure period, over and above the underlying time trend. Figures showing the estimated change in the month-on-month trends in statin initiation/cessation are in appendix part 7.

**Figure f1:**
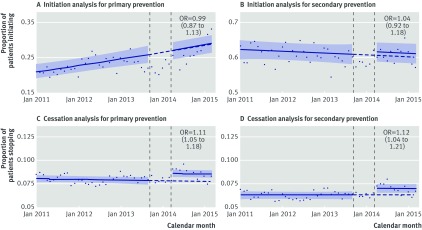
**Fig 1** Primary analyses evaluating step change in proportion of patients initiating and stopping statin for primary and secondary prevention of cardiovascular disease after exposure period (October 2013 to March 2014). Model used interrupted time series analysis with generalised linear model with binomial error structure, with break points at beginning and end of exposure period. Models allowed for change in level of proportion of patients initiating/stopping statin. Odds ratios therefore relate to relative change in odds of initiating/stopping statins after exposure period, in comparison with expected change based on pre-exposure predictions. In A and B denominators are patients with opportunity to initiate statin each month within study period, and numerators are patients who did initiate statin after indication. In C and D denominators are patients with statin prescription ending each month within study period, and numerators are patients who did not renew that prescription and hence were defined as stopping. Solid lines and shaded confidence intervals relate to linear predictions of log odds and 95% CI of event, respectively, calculated from model and converted into probabilities. Dotted lines are extrapolation of pre-exposure linear predictions of log odds converted to probabilities, to give hypothetical proportions of post-exposure period under counterfactual scenario of no changes after exposure

### Changes in statin initiation

There was no evidence of a stepped change in statin initiation for primary prevention after the exposure period compared with before, adjusted for the underlying trend over time (odds ratio 0.99, 95% confidence interval 0.87 to 1.13; fig 1 A), and no evidence of a stepped change in statin initiation for secondary prevention (1.04, 0.92 to 1.18; fig 1 B). When we looked at month on month trends in initiation over time, we estimated an underlying increase in initiation for primary prevention before the exposure period, which seemed to accelerate after the exposure period (appendix part 7a, P<0.001). We observed no change in the underlying trend in initiation for secondary prevention after the exposure period (appendix part 7b, P=0.41).

### Changes in statin cessation

Patients were more likely to stop taking statins after the exposure period compared with before, after we accounted for the underlying trend over time, for both primary and secondary prevention (odds ratio 1.11 (95% confidence interval 1.05 to 1.18) and 1.12 (1.04 to 1.21), respectively; fig 1 C/D). We found no evidence of any change in the underlying month on month trends in statin cessation (appendix part 7c and 7d, P=0.17 and P=0.16, respectively, for statins used for primary and secondary prevention).

### Stratified analysis

The increase in statin cessation after the exposure period seemed to vary by both duration of previous statin use and age group (fig 2). The increase in the likelihood of stopping after the exposure period was more pronounced among those who had taken statins for longer than those with shorter previous use (P<0.001 for trend in both primary and secondary prevention analyses). To aid comparability with other studies, we also divided duration of previous continuous prescription into less than and more than a year, and again we found a larger increase in the odds of stopping among those prescribed for longer (for cessation for primary prevention odds ratios were 1.10 (95% confidence interval 1.03 to 1.17) among those with less than one year of continuous prescription and 1.23 (1.15 to 1.32) among those with more than one year of continuous prescription; for cessation for secondary prevention the odds ratios were 1.10 (1.01 to 1.19) and 1.23 (1.13 to 1.33), respectively). The increased likelihood of stopping a statin used for primary and secondary prevention after the exposure period also became more pronounced in older age groups (P<0.001 for trend in both cases). We did not carry out stratified analyses for initiation as there were no measurable effects in the primary analyses.

**Figure f2:**
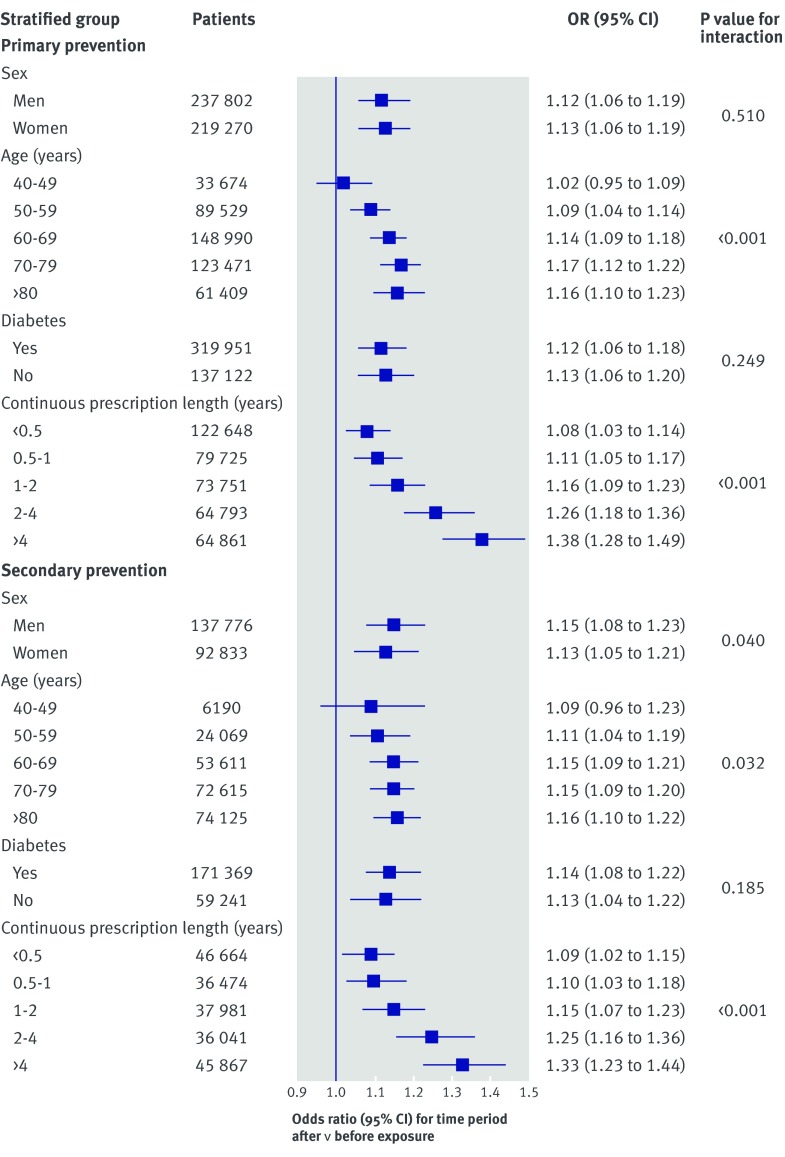
**Fig 2** Stratified cessation analyses evaluating step change in proportion of patients stopping statin for primary and secondary prevention of cardiovascular disease after exposure period (October 2013 to March 2014). Models used interrupted time series analysis with generalised linear model with binomial error structure, with break points at beginning and end of exposure period. Models allowed for change in level of proportion of patients stopping statin. Odds ratios therefore relate to relative change in odds of stopping statins after exposure period, in comparison with expected change based on pre-exposure predictions

### Post hoc analyses

In primary and secondary prevention, the increase in cessation seemed to be restricted to the first six months after exposure, after which cessation fell to a level similar to that expected based on pre-exposure trends for the next six months (fig 3). Consistent with this pattern, when we investigated both a step change and trend change simultaneously in a single model, there was evidence that immediately after the exposure period, patients were more likely to stop statins used for primary prevention (odds ratio 1.19, 95% confidence interval 1.02 to 1.39), followed by a month on month reduction back towards baseline (0.98 (0.97 to 0.99) per month). A similar pattern was seen for cessation of statins used in secondary prevention (1.25 (1.02 to 1.53) for immediate step change after exposure period and 0.98 (0.97 to 0.99) per month for subsequent month on month trend) (appendix part 8).

**Figure f3:**
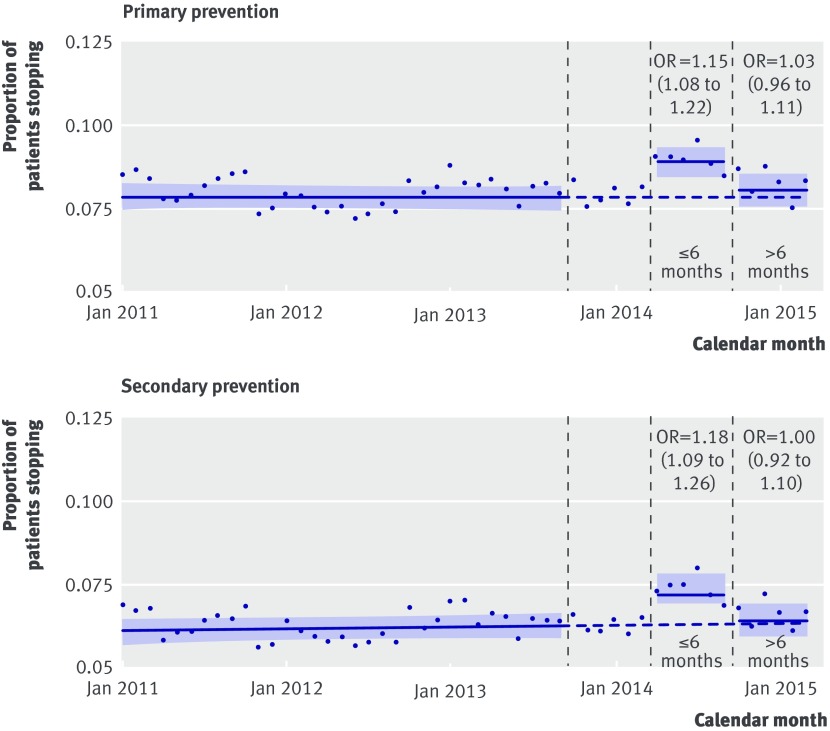
**Fig 3** Post hoc cessation analysis evaluating step change in proportion of patients stopping statin for primary and secondary prevention of cardiovascular disease, with post-exposure period stratified into ≤6 and >6 months. Denominators are patients with statin prescription ending each month within study period, and numerators are patients who did not renew that prescription and hence were defined as stopping. Models used interrupted time series analysis with generalised linear model with binomial error structure, with break points at beginning and end of exposure period. Models allowed for change in level of proportion of patients stopping statin. Odds ratios therefore relate to relative change in odds of stopping statins after for each 6 month section exposure period, in comparison with expected change based on pre-exposure predictions. Solid lines and shaded confidence intervals relate to linear predictions of log odds and 95% CI of event, respectively, calculated from model and converted into probabilities. Dotted lines are extrapolation of pre-exposure linear predictions of log odds converted to probabilities, to give hypothetical proportions of post-exposure period under counterfactual scenario of no changes after exposure

We also found evidence that patients were less likely to have any recorded risk score in the post-exposure period (odds ratio 0.85 (95% confidence interval 0.78 to 0.93) compared with pre-exposure); a similar pattern was seen for specific categories of risk score (fig 4).

**Figure f4:**
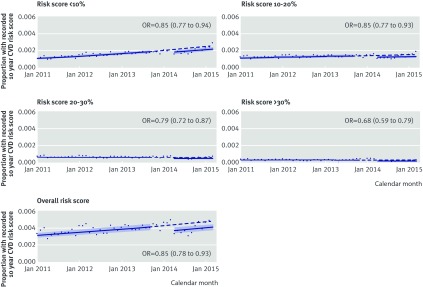
**Fig 4** Post hoc analyses evaluating step change in proportion of recorded 10 year risk scores for cardiovascular disease in each category after exposure period (October 2013-March 2014), using denominator of total number of patients under follow-up each month in CPRD. Denominators are all patients under follow-up in CPRD each month within study period, and numerators are patients that had recorded 10 year risk score for cardiovascular disease within each category in that month. Models used interrupted time series analysis with generalised linear model with binomial error structure, with break points at beginning and end of exposure period. Models allowed for change in level of proportion of patients with a recorded cardiovascular risk score. Odds ratios therefore relate to relative change in odds of having a recorded risk score after the exposure period, in comparison with expected change based on pre-exposure predictions. Solid lines and shaded confidence intervals relate to linear predictions of log odds and 95% CI of event, respectively, calculated from model and converted into probabilities. Dotted lines are extrapolation of pre-exposure linear predictions of log odds converted to probabilities, to give hypothetical proportions of post-exposure period under counterfactual scenario of no changes after exposure

### Negative control analysis

We found no evidence of changes in prescribing of treatment for glaucoma after the exposure period (appendix parts 9 and 10). When we moved the exposure period to 12 months earlier, the “post exposure” change in statin cessation rates disappeared as expected (odds ratio 1.01 (95% confidence interval 0.99 to 1.03) and 1.00 (0.98 to 1.03), respectively, for cessation in primary and secondary prevention).

### Impact on public health

We estimated that across the UK there was an excess of 218 971 patients who stopped taking a statin in the six months after the media coverage. When we applied a previously estimated restart rate of 66% among patients who stopped taking a statin without a statin related event,^28^ we calculated the number of excess cardiovascular events to be at least 2173 within the subsequent 10 years. Under the most pessimistic assumption that all the patients who stopped taking statins did so indefinitely, the estimated number of excess cardiovascular events rose to 6372.

## Discussion

### Key findings

A period of intense media coverage of statins and their side effects was followed by an increase in cessation of statins prescribed for both primary and secondary prevention of cardiovascular disease in UK primary care. This increase seemed to be temporary, and cessation had returned to expected levels after six months. We also identified that the tendency to stop was higher among patients who had used statins for longer and among older patients. Among those defined as newly eligible to receive statins, we did not observe a change in patients’ likelihood of initiating, over and above the underlying trend. Further investigation, however, showed a marked decrease in the proportion of patients having any cardiovascular disease risk score recorded after the media coverage, and hence a smaller pool of patients whose records met the criteria for initiation of statins for primary prevention.

### Findings in context of previous research

Our study is the first to attempt to quantify the effects of the UK media coverage of statins on prescribing in primary care and, to our knowledge, the first study in any country to look comprehensively at the effect of negative media coverage on rates of statin initiation and cessation, in both primary and secondary prevention of cardiovascular disease. A few recent studies have looked at specific aspects of media impacts on statin use. A study in Denmark reported that patients newly starting to take a statin were less likely to fill a second prescription if there were more negative statin related media stories in the period immediately after initiation.^15^ This is consistent with our findings that statin cessation rates are affected by negative news stories, and, importantly, our results suggest a similar effect is detectable even among those with longer established use. In Australia, Schaffer and colleagues reported a 28.8% (95% confidence interval 15.4% to 43.7%) increase in the discontinuation of statins in the week of the controversial TV programme *Catalyst*, which was deemed to be critical of statins.^13^ It should be noted that this refers to the peak increase in discontinuation at a weekly resolution, so is not comparable with our odds ratio estimates. In addition, the type, duration, and intensity of media coverage differed; nevertheless the results are qualitatively consistent with our findings. Kocas and colleagues reported that in Turkey, as news articles about statins increased from 2011 to 2013, the percentage of days covered by statins decreased (57% (interquartile range 8-83) in 2011, 58% (17-83) in 2012, and 50% (8-83) in 2013) (P=0.01).^16^ The overarching message is consistent with our study, but they focused on trends in statin adherence over a three year period. In France, Saib and colleagues also reported an increase in the number of patients that intended to stop statin therapy after media controversy,^14^ but their study was questionnaire based and no prescribing data were used, so, while again consistent with our findings, the results are difficult to compare directly with those in our study. Finally, consistent with our findings, a Dutch study by van Hunsel and colleagues used adverse drug reaction reports and found that, after a television programme about the benefits and risks of statins, there was a transient increase in the number of patients reporting reactions, a substantial proportion of whom also reported stopping treatment.^29^

Further studies have examined the impact of various other examples of health related media coverage on patients’ behaviour. A 2002 Cochrane review identified 15 mass media health interventions, and five studies of media coverage outside the context of a planned intervention (including coverage of breast cancer surgery for a public figure, side effects of drugs, and the disclosure of a sporting personality’s HIV status); all but one was associated with a change in health service use in the direction expected.^30^ Several more recent examples are also noteworthy. A New Zealand study found that media coverage about adverse events after a formula change in the thyroid drug Eltroxin (levothyroxine; GlaxoSmithKline) was followed by an increase in adverse event reports to a national medicines monitoring database, most markedly for the specific symptoms mentioned in television coverage.^31^ Data from Australia suggested that coverage of the singer and actress Kylie Minogue’s breast cancer was followed by a six week period in which self referral rates for breast cancer screening doubled among eligible women who had never previously presented for screening,^32^ ^33^ while UK celebrity Jade Goody’s diagnosis of cervical cancer was similarly associated with increased attendance for cervical screening,^34^ an increased incidence of referrals for colposcopy, and increased diagnoses of high grade cervical neoplasia.^35^

It is difficult to disentangle whether the changes in statin prescribing that we observed were driven by changes in the behaviour of physicians, patients, or both.^36^ It is noteworthy that we observed increases in cessation rates after the exposure period, but no corresponding decrease in statin initiation. The absence of a decrease in initiation rates for primary prevention, however, needs to be considered in the context of the observed decrease in the number of risk scores recorded after the exposure period, both overall and within risk strata, which indicates that the widespread debate and media coverage on risks and benefits of statins could have changed the general discourse between GPs and patients about management of risk of cardiovascular disease. It is possible that this was driven by a change in GP behaviour or by refusal of patients to engage with risk scoring for cardiovascular disease and discussions about statin use because of concerns about the potential side effects, which is a known worry for patients.^18^ Conversations between GPs and the most vocally concerned patients might have led to a reluctance to carry out risk scores because of the known worries surrounding the possible side effects of statins, leaving only those more likely to initiate in the denominator. These apparent changes in patterns of recording risk scores might also explain the unexpected acceleration in the already increasing trend in initiation of statins for primary prevention of cardiovascular disease among those with a recorded high risk score (appendix 7a). It is perhaps less surprising that there was no change in patients initiating statins for secondary prevention after the intense media coverage. After a potentially serious cardiovascular event, it is highly likely that a patient will be willing to take drugs proved to reduce their risk of having a recurrent event, regardless of critical media stories and potential side effects.

We observed an increasing tendency to stop taking statins as the length of previous continuous prescription increased. This might have been driven by immediate concerns about a recent indication for treatment (such as a cardiovascular event or high 10 year risk score) causing patients to be more reluctant to quit their treatment. In comparison, patients whose original indication occurred several years in the past might be comparatively comfortable to stop treatment after witnessing critical stories. Further qualitative research exploring attitudes among patients at different stages of statin use, however, would be needed to confirm this. Another possible explanation for apparent variation in the effect of media coverage is that particular groups of people might pay more attention to, or be more influenced by, health new stories; this might in particular explain why older patients were more likely to stop taking statins after the exposure period.

### Strengths and limitations

The CPRD is a large dataset and broadly represents the UK population,^21^ meaning our findings are generalisable to the wider population, and we were able to detect small effect sizes with great precision. We also believe that our populations will be similar to other developed populations as the indications for statins are broadly the same worldwide. Data were available for only a limited number of months after the exposure period, limiting the timescale over which we could detect effects, but as our analyses suggested that cessation rates were affected for only up to six months after the exposure period, this is unlikely to have been an important limitation.

Recording of prescriptions in the CPRD is automatic at the point of issue and therefore complete, though one limitation is that we could not be certain that prescriptions were subsequently dispensed at a pharmacy or taken by the patient. The actual proportions of patients initiating and stopping taking statins are likely to be subject to some measurement error as some patients will have initiated or restarted statins after the end of the predefined grace periods that we used. Also, if a patient began to self manage their prescription by taking a lower dose, it is possible that we could have incorrectly classified them as stopping statins because of longer than expected gaps between prescriptions. We believe that such error will be minimal because the grace periods were specifically selected such that in preliminary analyses only a small minority of patients went on to start (or restart) a statin after the selected times. Furthermore, our definitions for initiation and cessation remained the same throughout our study period, and it is unlikely that any measurement error would have changed after the exposure period and affected our main results. We did not capture use of low dose over-the-counter statins, which have been available since 2004, though it seems that uptake has been low,^37^ and there is no reason to think that the high level of media coverage would have led people to switch to over-the-counter statins.

Interrupted time series cannot confirm a causal link between the media coverage and the observed changes in the likelihood of stopping taking statins. The design avoids confounding by individual level factors such as smoking and obesity that are unlikely to vary over short term timescales, but it is possible that other external factors played a role in the observed changes. We carried out two negative control analyses in an attempt to exclude this, using both a different class of drug and a different time period, and we found no post-exposure changes in either of these analyses, strengthening our main finding. Nevertheless, it is still possible that other changes in the same time period, unrelated to the media controversy and affecting only statin use, could have driven the observed results.

If we assume causality, we estimated that increases in statin cessation due to the period of media coverage of side effects could result in at least 2173 excess cardiovascular events over 10 years, depending on the proportion of “stoppers” who re-started later. Our calculations were based on several assumptions and approximations and clearly could not take account of future changes in statin use and perceptions and other developments in prevention of cardiovascular disease. Varying assumptions also lead to substantial changes in the outcome, meaning these estimations should be interpreted with caution. We also cannot know from our data the extent to which patients were appropriately informed about the risk:benefit balance of statins and whether those who stopped would have been aware and accepting of the consequent increases in risk of cardiovascular disease. Patients can vary widely in the choices that they make about long term preventive drug treatment, and some choose not to take drugs that will extend their life.^38^ Finally, we did not attempt to take into account any possible benefits of stopping treatment with statins, which might have offset the increase in risk.

### Conclusion

Controversy over the risks and benefits of statins reported in both the medical and popular press was followed by a transient increase in patients stopping treatment prescribed for primary and secondary prevention. Additionally, a marked reduction in the proportion of patients receiving a risk score for cardiovascular disease suggests other important impacts on GP and/or patient behaviour. This research highlights the potential for widely covered health stories in the media to have an effect on real world behaviour related to healthcare and could be used to inform future interactions between clinicians, researchers, the academic press, and the wider media.

**What is already known on this topic**

Studies from Denmark, Australia, Turkey, and France have suggested that negative media stories can affect statin cessation and prescribing rates
Two controversial articles about statins were published in October 2013 in the UK, with a subsequent high volume of debate in the media about the associated potential risks and benefits

**What this study adds**

After the media coverage, there were no changes in statin initiation among those with a recorded new indication but an 11% and 12% increase in the likelihood of existing users stopping their treatment, for primary and secondary prevention, respectively
Across the UK these effects were estimated to result in over 200 000 patients stopping treatment with a statin in the six months after the media coverage
This research provides unique evidence describing the potential for widely covered health stories in the media to affect real world behaviour related to healthcare, with implications for public health, and has the potential to inform future interactions between clinicians, researchers, the academic press, and the wider media

## Web Extra

Extra material supplied by the author

## Notes

Ethical approval: The prespecified study protocol was approved by the Independent Scientific Advisory Committee for MHRA Database Research (ISAC), and the approved protocol including amendments is supplied in appendix part 11. Approval was also received from the London School of Hygiene and Tropical Medicine ethics committee.

Data sharing: The data were obtained from the Clinical Practice Research Datalink (CPRD). CPRD is a research service that provides primary care and linked data for public health research. CPRD data governance and our own license to use CPRD data do not allow us to distribute or make available patient data directly to other parties. Researchers can apply for data access at www.cprd.com, and must have their study protocol approved by the Independent Scientific Advisory Committee for MHRA database research (details at www.cprd.com/isac).

Transparency: The lead author affirms that the manuscript is an honest, accurate, and transparent account of the study being reported; that no important aspects of the study have been omitted; and that any discrepancies from the study as planned (and, if relevant, registered) have been explained.
