# Supplementary material for: Impact of statin related media coverage on use of statins: interrupted time series analysis with UK primary care data
Source: BMJ. 2016 Jun 28;353:i3283. doi: 10.1136/bmj.i3283 (PMC4925917; doi:10.1136/bmj.i3283)
Supplement: Supplementary file 1 — Appendix: Supplementary material [file mata031738.ww1_default.pdf]

Appendix: Supplementary material [posted as supplied by author]

Part 1 – Defining a recorded 10 year CVD risk score

All available recorded 10-year risk scores in the CPRD were identified during the study period. There were circumstances where more than one risk score per patient was recorded in the same day, which is believed to be due to a GP taking several blood pressure recordings and carrying out more than one calculation on that day. If this was the case, and there was a mixture of coronary heart disease risk scores and cardiovascular disease risk score, then the cardiovascular disease risk score(s) was used. If there were still more than one recorded risk score, and they fell in conflicting risk score categories, then none of the patients' records were used as we were unable to identify which category the true score belonged to. If there were several recorded risk scores on the same day, in the same risk score category, this risk score category was assigned to the patient. For primary analyses assessing the initiation of statins for primary prevention, we restricted to patients with a risk score ≥20% because such patients were eligible for statin treatment both before and after the July 2014 NICE guideline changes. A patient could also have more than one CVD risk score and therefore more than one opportunity to initiate a statin for primary prevention (in which case they would contribute to more than one monthly data point).

Part 2 – Defining the grace period for initiation analysis in patients prescribed statins for secondary prevention

We identified all incident cardiovascular events between January 2011 and October 2013 and calculated the length of time between date of cardiovascular event and incident statin prescription. We identified that 59% of all patients with an incident cardiovascular event in this period had been prescribed a statin in primary care within 60 days of the event. Taking into account that defining an extremely long grace period could result in identifying statin prescriptions which were not related to the cardiovascular event in question, we deemed this an adequate proportion of patients initiating statins, and hence defined the grace period to be 60 days.

Part 3 – Defining the length of statin prescription

For all statin prescriptions, if the daily dose was of an unreasonable value (greater than 4 or 0 tablets per day), the median of one tablet per day was imputed. If the quantity of tablets prescribed was of an unreasonable value (greater than 300 or less than 7 tablets), then the median of 28 was imputed. In the event where a patient had more than one statin prescription on the same day, the length of all prescriptions were summed to calculate the overall prescription length.

Part 4 – Google search term trend analytics using the search term `statin side effects’

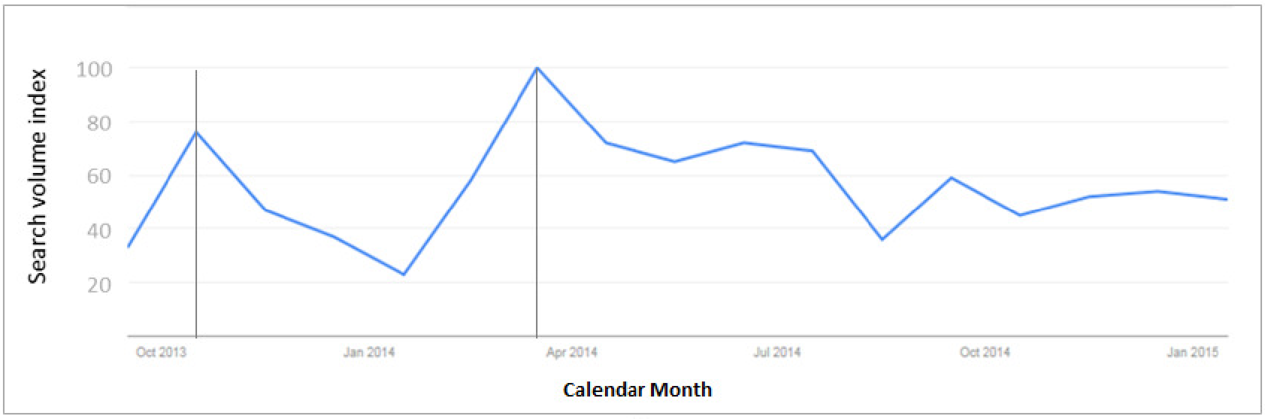

[1]The month with the highest search volume within the specified period volume has a search volume index of 100 and all other months are assigned an index with relation to this.

Part 5 - Negative control analysis detail: drugs used for glaucoma

Two analyses were carried out: one for cessation and one for initiation analyses. For the cessation analysis, for each calendar month, we identified all individuals aged $\geq$40 years and in receipt of a glaucoma drug prescription that ended within that calendar month. Prescription end dates were calculated as 28 days after the prescription start date. A patient could be included in more than one monthly cohort if they had multiple prescriptions ending during the study period. The grace period was calculated using the same criteria as the preliminary analysis for the main cessation analysis. All glaucoma prescriptions between January 2011 and October 2013 were identified and the length of time between the end of prescription and the start of a new prescription was calculated. We identified that 90% of prescriptions were followed up with a new prescription within 46 days of the initial prescription ending, which was then used as the grace period.

For the initiation negative control analysis, we calculated the proportion of all patients under follow-up in the CPRD that month who had an incident glaucoma drug prescription. For both analyses, we used the same statistical methods used for primary analyses and evaluated a step and trend change after the exposure time period, in comparison to before.

Part 6 - Public health impact calculation

The excess number of patients that stopped statins in the six month period following our exposure time period was 15109. This was calculated by comparing the monthly modelled proportions of patients stopping within this six month period using our post-hoc analyses, with the hypothetical monthly proportions in the same period under the counterfactual scenario of no changes after exposure, for both primary and secondary prevention. Each monthly proportion difference was then multiplied by the total number of patients in the denominator that month. These totals were then summed over the six month period for both primary and secondary prevention models to calculate the total number of “excess stoppers”. We then estimated the number of excess CVD events among these patients over the following 10-years, based on the assumptions outlined below, and finally we scaled this up to reflect the likely impact in the full UK population.

1) CVD risk

There were 84,884 distinct patients that stopped statins and were originally prescribed for primary prevention in the 6 months following the exposure time period. 34,997 of these had previously had a recorded 10-year CVD risk score in CPRD, and the median 10-year risk was at the upper end of the 10-20% 10 year CVD risk category. Hence, we decided to assume a 10-year CVD risk of 20% among patients who stopped statins used for primary prevention. Patients with a prior CVD event are usually considered at "clinically high risk" of a further CVD event, based on clinical trial data in secondary prevention populations where five year CVD event rates of over 20% are regularly reported [28, 29]. For a conservative estimate, we therefore also assumed a 10-year CVD risk of 20% to the number of patients that stopped statins used for secondary prevention.

2) Risk reduction attributed to statins

Using results from the CTT, the relative risk reduction of a major vascular event in patients prescribed statins in comparison to a non-statin prescribed control was 19% (95% CI: 12%,23%), for all patients with a 5 year major vascular event risk of ≥20% and <30%.

3) Proportion that would have stopped later, regardless of the media coverage

A proportion of patients stopping due to the media coverage would have stopped therapy at some later point in their subsequent 10-year follow-up, regardless of the media coverage. To provide an estimate of the proportion of patients stopping statins within a 10-year period, we followed up a cohort of patients with a statin prescription ending in March 2003, for 10 years. We defined an event as no further prescriptions for at least a year following the end of a prescription, or death within a year following end of their last prescription. Patients were censored if they left CPRD within a year of the end of their last prescription, or after 10 years if they were still being prescribed statins at that date. Kaplan-Meier methods were then used to analyse time to statin cessation, and at 10 years the survival function (estimated proportion remaining alive and on a statin) was 0.511. Among patients identified as stopping or dying during follow-up, the median time to event was 5.21 years.

Final calculation of excess CVD events due to media coverage

The 10-year CVD risk and statin-associated risk reduction were then applied to the total number of excess stoppers. Based on the analysis described above, we assumed that even in the absence of the media coverage, only 51.1%% of these patients would have continued statins for the full 10 years, and the remaining 48.9% would have received only 50% of the protection (based on median of 5.21 years use out of 10). The final estimated number of CVD events attributed to the media coverage among CPRD patients was then scaled up to the total population of the UK (CPRD covers 6.9% of the total UK population) to calculate the excess number of CVD events within the UK attributed to the media controversy.

Post cessation restart rates

The above calculation assumed that those stopping their statin never restarted. However, Zhang et al. recently reported that 65.9% of patients who stopped statins, without a statin-related event, had another prescription over the subsequent 12 months. To provide a second more conservative estimate allowing for this, we therefore repeated the above calculation assuming that 65.9% of the 15109 excess patients who stopped statin therapy restarted quickly and had no loss of protection.

Part 7 – Primary analyses evaluating a trend change in the proportion of patients initiating and stopping statin therapy for primary and secondary prevention of CVD after the exposure time period (October 2013 – March 2014)

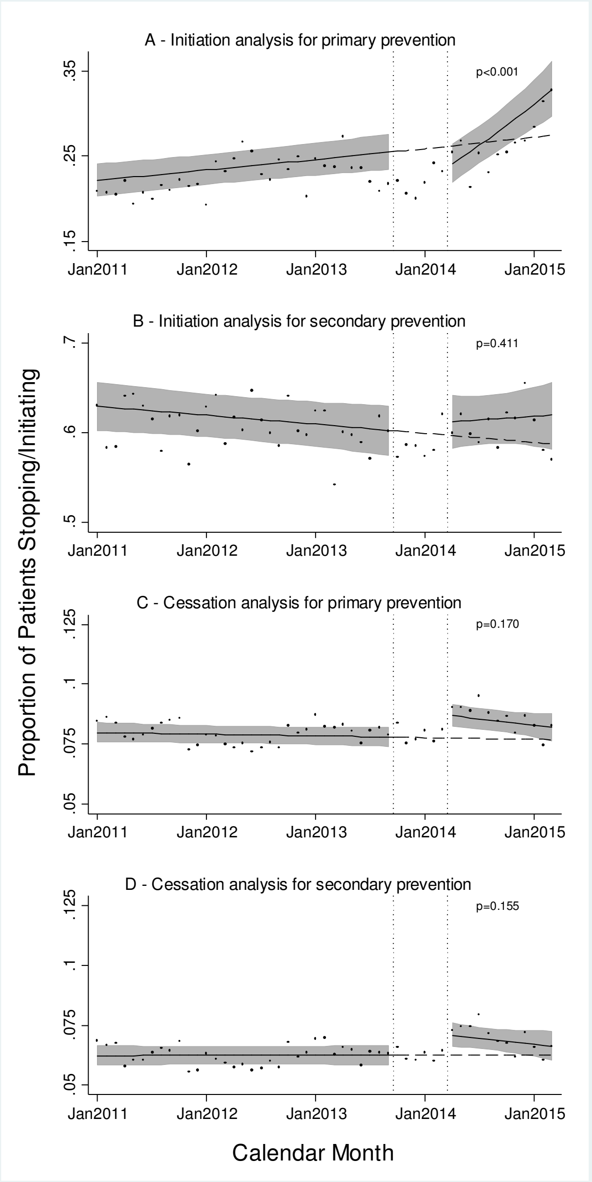

[1]An interrupted time series analysis using a generalised linear model with a binomial error structure was used for all models, with break points at the beginning and end of the exposure time period. Models allowed for a change in trend of the proportion of patients initiating/stopping statin therapy. P-values relate to the Wald test comparing the trend of initiating/stopping statins before the exposure period, to the trend after.

Graphs A and B: Denominators are the patients with the opportunity to initiate statin therapy each month within the study period, and numerators are the patients that did initiate statin therapy following the prior indication.

Graphs C and D: Denominators are the patients with a statin prescription ending each month within the study period, and numerators are patients that did not renew that prescription and hence were defined as stopping.

Part 8 – Post-hoc analysis evaluating a step and trend change in the proportion of patients stopping statin therapy for primary and secondary prevention of CVD after the exposure time period (October 2013 – March 2014)

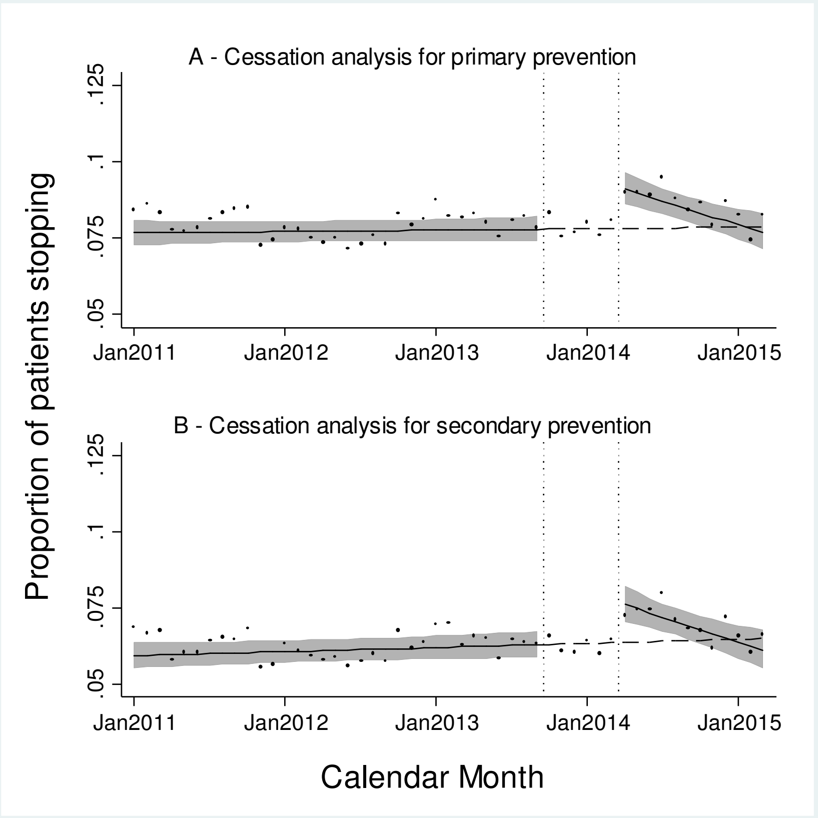

An interrupted time series analysis using a generalised linear model with a binomial error structure was used for all models, with break points at the beginning and end of the exposure time period. Models allowed for both a change in level and a change in trend of the proportion of patients stopping statin therapy.

Denominators are the patients with a statin prescription ending each month within the study period, and numerators are patients that did not renew that prescription and hence stopped.

In all graphs, the solid lines and shaded confidence intervals relate to linear predictions of the log odds and 95% confidence

Part 9 – Control analysis evaluating a step change in the proportion of patients initiating and stopping glaucoma therapy after the exposure time period (October 2013 – March 2014)

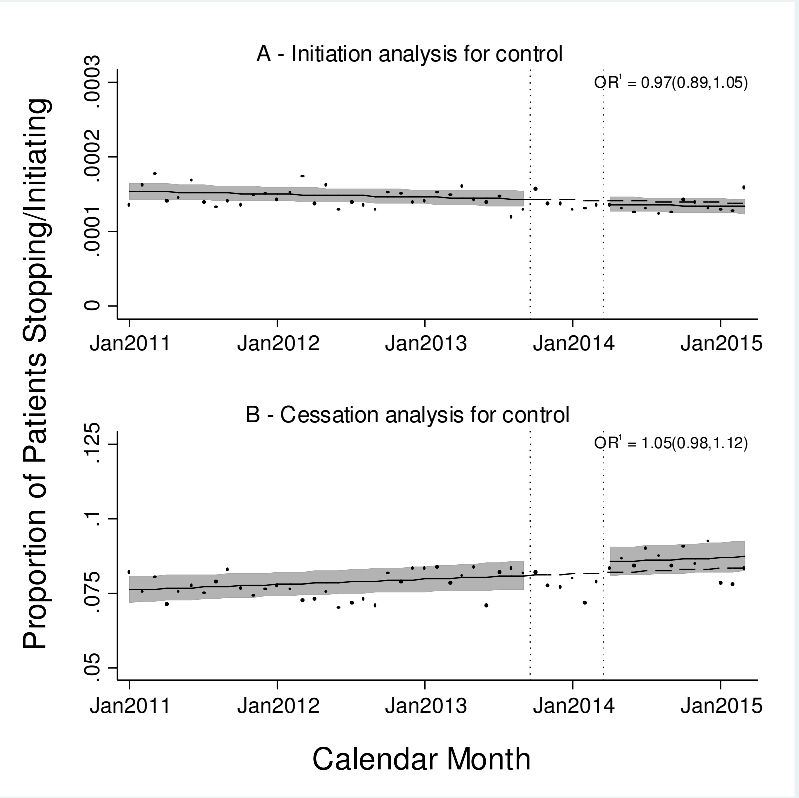

[1]An interrupted time series analysis using a generalised linear model with a binomial error structure was used for all models, with break points at the beginning and end of the exposure time period. Models allowed for a change in level of the proportion of patients initiating/stopping statin therapy. ORs therefore relate to the relative change in the odds of initiating/stopping statins after the exposure time period, in comparison to what we expected based on pre-exposure predictions.

Graph A: Denominators are the all patients under follow up in the CPRD each month within the study period, and numerators are the patients that initiated glaucoma therapy.

Graph B: Denominators are the patients with a glaucoma prescription ending each month within the study period, and numerators are patients that did not renew that prescription and hence were defined as stopping.

In all graphs, the solid lines and shaded confidence intervals relate to linear predictions of the log odds and 95%

Part 10 – Control analysis evaluating a trend change in the proportion of patients initiating and stopping glaucoma therapy after the exposure time period (October 2013 – March 2014)

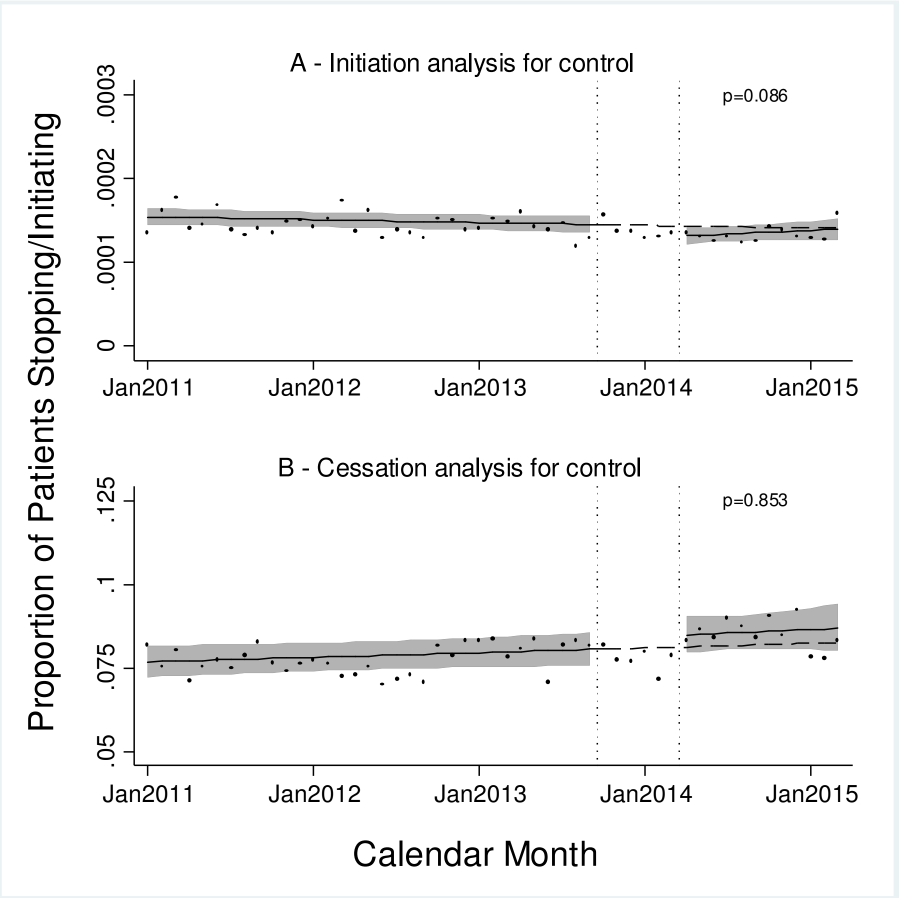

[1]An interrupted time series analysis using a generalised linear model with a binomial error structure was used for all models, with break points at the beginning and end of the exposure time period. Models allowed for a change in trend of the proportion of patients initiating/stopping statin therapy P-values relate to the Wald test comparing the trend of initiating/stopping statins before the exposure period, to the trend after.

Graph A: Denominators are the all patients under follow up in the CPRD each month within the study period, and numerators are the patients that initiated glaucoma therapy.

Graph B: Denominators are the patients with a glaucoma prescription ending each month within the study period, and numerators are patients that did not renew that prescription and hence were defined as stopping.

Part 11 – ISAC Protocol

Impact on initiation and cessation of statins amidst the media interest in the side effects of lipid lowering therapy prior to the 2014 NICE lipid modification guidelines

Lay Summary

In July 2014, the National Institute for Healthcare and Clinical Excellence (NICE) amended their guidelines for statin therapy for the primary prevention of cardiovascular disease, lowering the cardiovascular risk threshold for which a patient should be recommended statins from 20% to 10% ten year risk. In the context of debate surrounding these impending changes, Abramson et al. published a paper in October 2013 claiming that prescribing statins to people with a low risk of cardiovascular disease would increase the number of adverse events, without providing overall health benefit. A high volume of media coverage followed.

We would like to examine if media coverage around adverse side effects impacted the initiation and cessation of statin prescribing in patients at high risk of a cardiovascular event in the UK.

We will do this by calculating the percentage of patients starting and stopping statins for both primary and secondary prevention at monthly intervals from 2011-2015. We will then investigate changes in the proportion of patients starting and stopping at key time points.

Background

Statins are lipid lowering drugs that reduce the risk of cardiovascular disease [1]. In the 12 months preceding March 2008 45.2 million statins prescriptions were dispensed in primary care in England at a cost of £483.36 million [2].

In July 2014, NICE published amended guidelines in relation to patients that should be recommended a statin prescription [3]. The amendments stated that patients whose 10-year risk of cardiovascular disease is over 10% should be prescribed statins. Previous guidelines published in 2007 recommended a risk cut off point of 20%. The new guidelines also recommended the exclusive use of the QRISK2 method for calculating cardiovascular disease risk and continued to recommend the prescription of statins to all patients with existing cardiovascular disease. There was much controversy prior to the publication of these guideline changes, especially since there was found to be a wide variation between practices in statin prescribing to patients at higher risk ($\geq$ 20%) of cardiovascular disease [4]. For example, Abramson et al. published a paper in the BMJ in October 2013 claiming that broadening the recommendations on cholesterol lowering guidelines to include statin therapy for low risk individuals will unnecessarily increase the incidence of adverse effects, without providing overall health benefit [5]. More specifically, Abramson et al included a claim (later the subject of a correction) that side-effects occurred in approximately 18-20% of patients using statins. This created concern as it was thought that high risk patients may have stopped statin therapy as a result of these comments, even though the controversy was stimulated by arguments about treating low risk patients. A high volume of media interest followed.

Although there have been anecdotal suggestions from a questionnaire by the British Cardiovascular Society [6], there is no quantitative evidence as to whether this controversy around potential side effects of statins has impacted the initiation and cessation of the drug within UK general practice.

Objective, specific aims and rationale

Objective

The overall objective is to estimate changes over time in initiation and cessation of lipid-lowering therapy for both primary and secondary prevention, and the impact of media interest in side effects.

Aims

1. To evaluate time trends in initiation of statins in patients with and without incident cardiovascular events from 2011-2015, and whether the proportion initiating has been affected by media controversy about side effects;
2. To evaluate time trends in cessation of statins in patients with and without existing cardiovascular disease from 2011-2015, and whether the proportion stopping has been affected by media controversy about side effects.
3. To investigate whether any such media effects differ by patient characteristics, cardiovascular risk factors, or overall cardiovascular risk.
4. To estimate the public health impact of any changes in statin usage patterns due to the media controversy.

Rationale and public health importance

Following the controversy surrounding potential side effects of statins, it is possible that patients eligible for statins have declined the drug, and that some existing users have stopped treatment. As statins have been proven to reduce the risk of cardiovascular disease, such patients are at increased risk of a cardiovascular event.

Study type

Hypothesis testing. The null hypothesis is that the controversy in the media following the comment by Abramson et al that the statin side effect rate is 18-20% did not affect the initiation and cessation of statin therapy in UK general practice between 2011 and 2015.

Study design

Interrupted time series analysis of trends of initiation and cessation of statins.

Linked Data

No linked data are required for this study.

Study populations

Initiation analyses: Registered patients aged 40 and over with documented eligibility to receive a statin for primary (i.e. cardiovascular risk of ≥20% over ten years) or secondary prevention of cardiovascular disease.

Cessation analyses: Registered patients aged 40 and over receiving statins for primary or secondary prevention of cardiovascular disease.

Exposures, outcomes and covariates

Exposures

We aim to describe trends in both initiation and cessation of statins over time. We also specifically want to investigate whether there are significant changes in trends of initiation and cessation surrounding the following time points:

Primary time exposure

- Media coverage regarding the side effects of statins between November 2013 and March 2014 – The aforementioned article by Abramson et al discussing the adverse side effects of statins was published in the BMJ on 22/10/2013. We have also identified, using google trends analytics, that peak public interest in the side effects of statins was within March 2014 [Fig. 1]. This is based on the google search term 'statin side effects' and corresponds to the volume of people using this search term. We therefore intend to investigate the trends in initiation and cessation of statins before, during and after the period between these two events (October 2013 – March 2014). We decided on this exposure time period as controversy surrounding statin side effects first came into public interest at the time of the Abramson BMJ paper. This information was then publically available with interest reaching its peak within March 2014 when most major national news media (including the BBC and national newspapers) covered the subject. Therefore, all patients with a vested interest in statins are likely to have been exposed to the information at some point between November 2013 and March 2014 inclusive. We will subsequently carry out a sensitivity analysis shifting the exposure time period, hence assessing a time lag in the patients reacting to the media coverage.

Figure 1: Google analytics search term trends using the search term 'statin side effects'

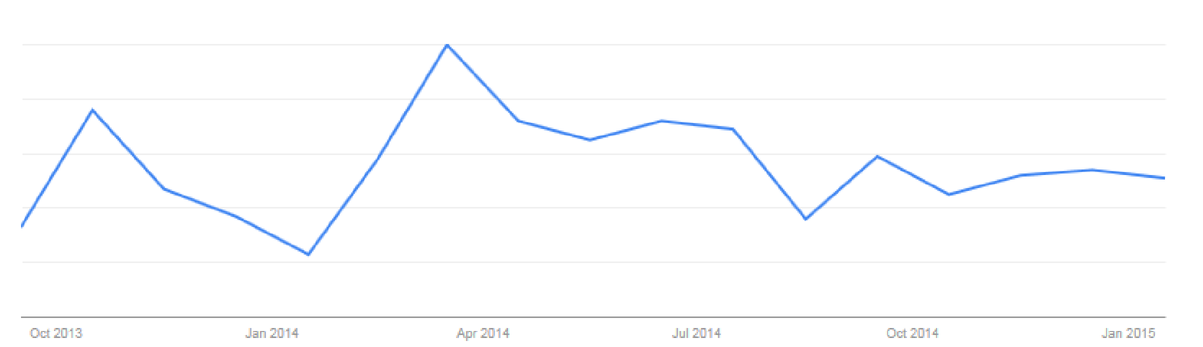

Outcomes

- Initiation of statins will be defined as a first recorded statin prescription at least 1 year after start of follow-up in CPRD. All patients whose first prescription is up to a year after registration will be excluded as this could be a continuation of an existing prescription from a previous GP
- Cessation of statins among those on a statin at a particular time point will be defined as having no further statin prescriptions within a specified grace period (identified in preliminary analyses defined below) following the end date of last prescription.

Key Definitions

Cardiovascular event

An event relating to coronary heart disease (myocardial infarction, angina and revascularisation procedures), cerebrovascular disease (stroke, transient ischaemic attack), or peripheral vascular disease (abdominal aortic aneurism and intermittent claudication). Codes relating to incident events and pre-existing conditions will be identified, based on codelists developed for the CALIBER project (Cardiovascular Disease Research Using Linked Bespoke Studies and Electronic Records, https://www.caliberresearch.org/).

Cardiovascular risk scores

All available methods of risk score calculation will be identified. A pre-specified code list for cardiovascular risk score events is available in appendix 1. To minimise the effect of actual guideline changes on our results (and hence focus on the effects of media controversy), we will restrict to those with calculated risk scores of over 20%, or a Read term which indicates a ten year cardiovascular risk of 20% or more; such patients were eligible for statin treatment both before and after the July 2014 guideline changes.

Statins

Prescriptions in relation to all forms of statin will be identified. The duration and prescription end date of each prescription will be calculated using the number of tablets prescribed and daily dosage instructions; where this information is unavailable, we will use the median prescription duration. The end date will be calculated as the date of prescription plus the number of days' drug supply.

Data/Statistical Analysis

All data will be analysed using Stata v13 (StataCorp, Texas)

Preliminary analysis

1. *Analysis of prescription gaps to inform operational definition of continuous statin use*: For all statin prescriptions from Jan 2011 to Oct 2013 (so that these preliminary analyses aren't affected by the

media controversy) we will calculate the gap between the end of one prescription and the start of the next, and plot the distribution of gaps. We will use this analysis (without reference to the main time trends of interest) to inform the gap, or 'grace period' that should be used to deem a prescription continuous. Any prescription gaps exceeding our defined grace period will be defined as a cessation in statin prescription, with the date of cessation being the end date of the most recent prescription.

2. *Preliminary analysis to inform definition of initiation for secondary prevention*: It is likely that a patient is admitted to hospital after a cardiovascular event. Further to this, it is likely that they are administered statins whilst in hospital and given statins on discharge. These hospital-based prescriptions would not be picked up in the primary care data we are using, so in evaluating statin initiation among these patients, there is a need to allow for a delay until the patient has the opportunity to receive their first prescription from a GP. In order to understand how long a delay is necessary, we will plot the distribution of the number of days between incident cardiovascular events and first statin prescription in primary care for all incident cardiovascular events between Jan 2011 and October 2013. We will use this to define a sensible length of time that patients should be followed up to determine if they initiated statins in primary care after an incident cardiovascular event.

Aim 1 – Initiation of statins

(i) Initiation for secondary prevention

For each calendar month between 2011 and 2015, we will identify all patients experiencing a first cardiovascular event during the given calendar month (denominator), and we will calculate the percentage of these patients initiating a statin based on receiving a first statin prescription within a pre-specified length of time since the cardiovascular event (as determined by the preliminary analyses described above, Figure 2). Thus, for each calendar month, we will generate a single proportion of patients that initiate statins as per the guidelines, which will then be plotted by calendar month from 2011-2015. All patients whose CPRD follow-up ends during the pre-specified time period used to capture statin initiation will be excluded, since it would be impossible to reliably ascertain initiation for these individuals.

Figure 2: Explanation of cohort for initiation of statins in secondary prevention

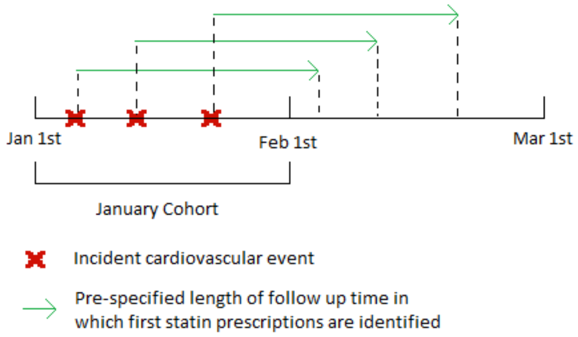

Modelling changes in proportion of patients initiating statins by calendar time

We will perform an interrupted time series analysis, using segmented linear regression, of proportion of patients initiating statins each month. We will divide time into segments before, during and after the exposure time period (October 2013-March 2014, Figure 3) [7,8]. We will then explore a change in trend of

initiation at the beginning and end of the exposure time period by testing for a change in slope at these time points. We will also explore a change in trend comparing the time period before exposure to the time period after exposure. As a secondary analysis, we will also explore a step-change in initiation by testing for a change in level. We will include a term in the regression model for the lagged residuals, to account for autocorrelation (observations closer together in time series tend to be more similar than observations further apart), and we will assess for seasonality, adjusting accordingly if necessary [9].

Figure 3: Illustration of model for initiation time trends in relation to key time points

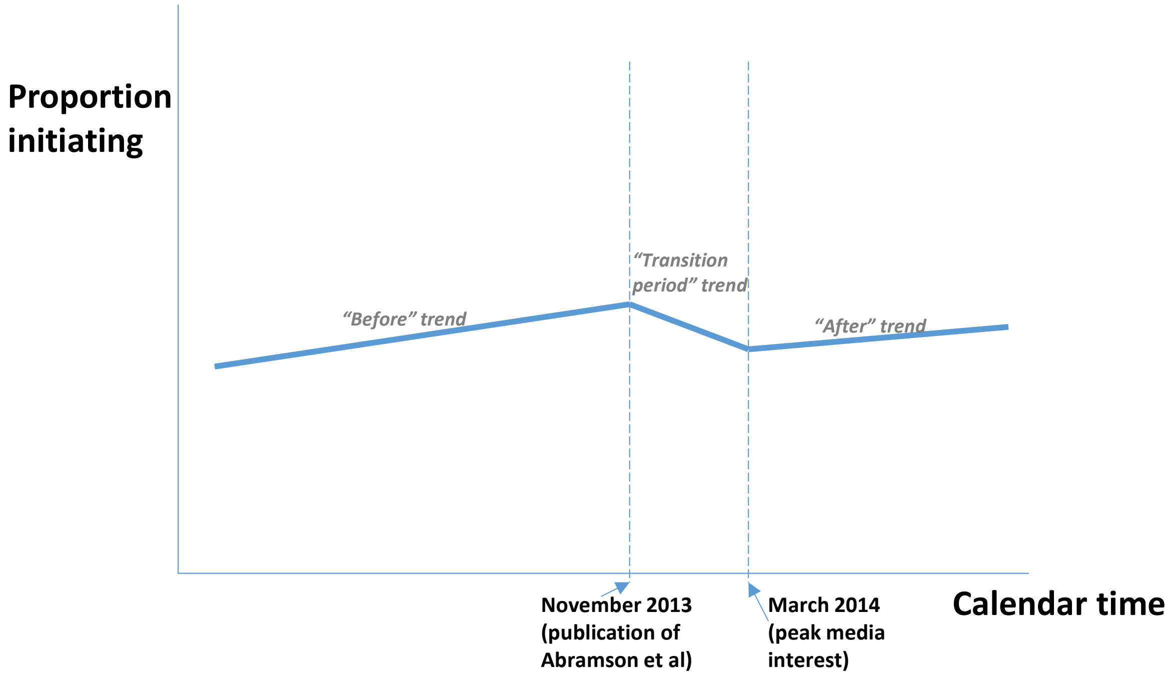

(ii) Initiation for primary prevention

For each calendar month between 2011 and 2015, we will identify all patients with a calculated score of ≥20% ten year cardiovascular risk, with no previous cardiovascular events, during the given calendar month (denominator). We will calculate the percentage of these patients initiating a statin within 1 month. The 20% risk threshold will be used because eligibility for primary prevention among this group has not changed since 2011, so the pre- and post-exposure comparison will not be complicated by the 2014 guideline change. All further analyses will be carried out as in Aim 1 (i), but patients with a cardiovascular event within the one month follow up, which pre-dates any statin prescription, will also be excluded as these patients will be identified in Aim 1 (i). We will also explore effect modification by age, sex and cardiovascular risk in stratified secondary analyses.

Aim 2 – Cessation of statins

(i) Cessation among those taking statins for secondary prevention

For each calendar month between 2011 and 2015, we will identify (as the denominator) all patients with existing cardiovascular disease and a statin prescription which ends during that month (where end date is the date of last prescription plus number of days' drug supply); this represents all patients with an opportunity to stop their statin that month according to our definition of stopping (namely not renewing a prescription that has run out, within a pre-defined grace period). If a patient has several prescriptions which end in the same month, then their latest prescription end date will be used.

The numerator will be the number of patients that stop taking statins, defined as no further prescriptions within the grace period defined in our preliminary analyses (Fig 4).

The proportion of patients stopping will then be plotted by calendar month from 2011-2015. All results will be stratified by length of time prescribed to a statin without stopping (<12 months, 12-36 months, >36 months).

Figure 4: Explanation of cohort for cessation of statins in secondary prevention

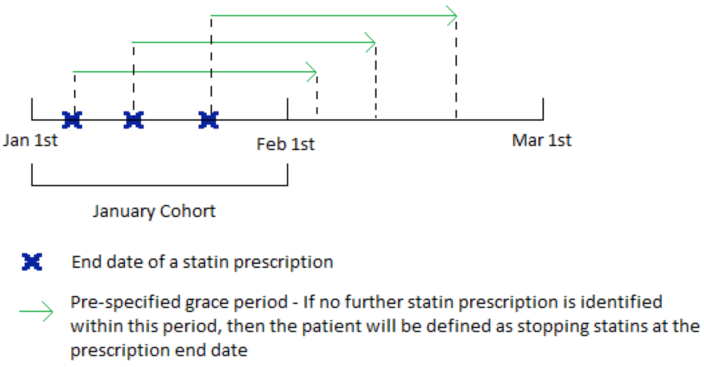

Modelling changes in proportion of patients that stop taking statins by calendar time

We will then use segmented linear regression for the interrupted time series analysis, using the same exposure time period and approach as in Aim 1.

(ii) Cessation among those taking statins for primary prevention

For each calendar month between 2011 and 2015, we will identify all patients without a previous cardiovascular event and a statin prescription with an end date within that month (denominator). Cessation of statin prescribing will be defined as in Aim 2(i). Percentage of patients that stop taking statins will then be plotted by calendar month from 2011-2015 and all further analyses will be carried out as in Aim 2 (i). However, patients with a cardiovascular event during the predefined grace period will also be excluded from the denominator.

Control Analysis

In order to assess if a possible significant step-change or change in trend in initiation and persistence to statins is due to exposure of peak public interest in statin side effects, we will run the same analyses using a

negative control. For initiation and persistence in secondary prevention we will use patients prescribed beta blockers after a diagnosis of myocardial infarction, and for initiation and persistence in primary prevention we will use patients exposed to an ACE inhibitor or angiotensin 2 receptor blocker as a first line treatment for newly diagnosed hypertension. The same primary and secondary time exposure points will be used. We have no reason to expect any significant changes in usage patterns for these drugs at the pre-specified exposure time points.

Aim 3 – effect modification

To explore whether statin initiation/cessation are more or less affected by media controversy among key subgroups, we will also conduct stratified analyses by age group, sex, risk score stratum, diabetes status, and treated hypertension status, as well as duration of statin use (for cessation analyses).

Aim 4 – public health impact

To estimate the potential public health impact of changes in statin initiation and prescribing trends, we will compare the observed proportion of individuals initiating and stopping a statin in each month after March 2014, with the hypothetical proportions of people initiating/stopping in the same period under the counterfactual scenario of no changes in trends at the two time points of interest (i.e. simply projecting the modelled “before trend” line [see Figure 3] forward), thus estimating the proportion of people that may have declined to initiate, or stopped a statin, due to the media controversy, under the assumption of causality. We will combine this calculation with national cardiovascular disease/mortality event rates and known statin efficacy estimates from the Cholesterol Treatment Trialists’ Collaboration [10] to estimate the impact of the changes in statin usage patterns on number of cardiovascular disease events and deaths. For simplicity, this analysis will initially assume that those declining to initiate in a given calendar month do not go on to initiate later, and that those stopping do not later restart. Since this is a strong assumption, we will also examine actual statin usage patterns among those who do not immediately initiate, or who cease therapy, and if a substantial proportion start (or restart) therapy later, we will repeat the impact analysis allowing for this.

Sample size/power calculation

The following sample size calculations are based on an extract of 1,000,000 patients from the CPRD, scaled up to represent the 14,000,000 patients in the full sample. Based on our inclusion and exclusion criteria, the individual data points estimating monthly proportions of patients initiating and ceasing statin therapy will include ≥1,148 patients eligible for the statin initiation analyses, and ≥146,622 patients eligible for the cessation analyses, leading to high precision of the individual data points, with confidence intervals ranging from ±0.20% to ±2.89%.

For the interrupted time series analysis we will have at least 48 data points (monthly initiation and cessation proportion estimates), with 10 data points after our exposure time period (using the July 2015 CPRD build) and each data point will have at least 336 outcome events. Using a power calculation for the comparison of Poisson counts across two groups, we will have 90% power at a 95% confidence level to detect a rate ratio of at least 1.07 when comparing the rate of either initiation or cessation before and after the exposure time period

Patient/User group involvement

We have not engaged with patients or user-groups in the development of this protocol.

Limitations of the study design, data sources and analytic methods

Missing data

There is likely to be missingness in the variables used to report cardiovascular risk score, even when there is a Read code which implies a calculation has been made. Feasibility analyses suggest the missingness will be approximately 11%. Entries will be excluded if they have a missing risk score calculation and the risk stratum cannot be determined from the Read term.

Random error

The study will be conducted among all currently registered patient fulfilling the exclusion and exclusion criteria. We estimate between 1,100 and 250,000 acceptable patients for each calendar-month-specific cohort, so random error will be minimal.

Bias and generalisability

GP practices are self-selecting with respect to their contribution to CPRD, however those practices contributing are thought to be broadly representative of the UK population. Very few patients within contributing practices refuse to participate at an individual level and this is not thought to bias the results in any way. Therefore our analyses should reflect the distribution of statin prescriptions among the UK population, and should be generalisable.

The analyses of statin initiation require a minimum period of follow-up to assess whether a statin was started. It is possible that those not initiating a statin and therefore without the protection offered by this treatment, may be less likely to reach the end of this minimum follow-up period, due to a higher risk of death. This would result in statin initiation rates being overestimated. We do not think this is a major limitation because the minimum follow-up periods are short (e.g. 1 month for the primary prevention analysis), and even if overall initiation rates are overestimated, this should not affect the main calendar time trends of interest.

Plans for disseminating and communicating results

The study findings will be submitted for publication in peer-reviewed scientific journals, and will be presented at appropriate conferences and other meetings. We will engage fully in opportunities to communicate our results to the general public, including via the media.

ISAC References

1. Taylor, F, et al. Statins for the primary prevention of cardiovascular disease. Cochrane Database Syst Rev, 2013. 1: p. Cd004816
2. National Institute for Health and Clinical Excellence. NICE implementation uptake report: statins for the prevention of cardiovascular events. NICE technology appraisal 94. 2008: London
3. National Institute for Health and Care Excellence (NICE). Lipid modification: cardiovascular risk assessment and the modification of blood lipids for the primary and secondary prevention of cardiovascular disease. NICE clinical guideline 181. 2014: London.
4. van Staa, T-P, et al. The efficiency of cardiovascular risk assessment: do the right patients get statin treatment? Heart 2013;0:1-6.
5. Abramson, et al. Should people at low risk of cardiovascular disease take a statin? BMJ. 2013. 347:f6123.
6. BCS ‘Statins and the Media’ Survey Results. British Cardiovascular Society. 2014.
7. Wagner, AK, et al. Segmented regression analysis of interrupted time series studies in medication use research. Journal of Clinical Pharmacy and Therapeutics. 2002. 27. 299-309.
8. Morgan, OW, et al. Interrupted Time-Series Analysis of Regulations to Reduce Paracetamol (Acetaminophen) Poisoning. PLoS Medicine. 2007. 4. e105.
9. Bhaskaran, K, et al. Time series regression studies in environmental epidemiology. International Journal of Epidemiology. 2013;42:1187–1195
10. Cholesterol Treatment Trialists' (CTT) Collaborator. The effects of lowering LDL cholesterol with statin therapy in people at low risk of vascular disease: meta-analysis of individual data from 27 randomised trials. Lancet 2012; 380(9841): 581-90

ISAC Appendices

Appendix 1 – Cardiovascular risk score code list

medcode	readterm
7913	Coronary heart disease risk
10128	Cardiovascular event risk
10302	Framingham coronary heart disease 10 year risk score
13283	Coronary heart disease risk
18581	Low risk of primary heart disease
18948	Moderate risk of primary heart disease
19165	Goldman cardiac risk index
22210	High risk of primary heart disease
24721	Framingham coronary heart disease 10 year risk score
25486	Framingham coronary heart disease 5 year risk score
26627	At risk of heart disease
26815	Framingham coronary heart disease 5 year risk score
29433	High risk of heart disease
36908	UKPDS 10yr coronary heart disease risk score
43934	Joint British Societies cardiac risk score
43938	Framingham coronary heart disease 10 yr adjusted risk score
55103	JBS cardiovascular disease risk 10-20% over next 10 years
55104	JBS cardiovascular disease risk <10% over next 10 years
55105	JBS cardiovascular disease risk >30% over next 10 years
55109	JBS cardiovascular disease risk >20% up to 30% ov next 10 yr
71748	Coronary heart disease risk clinical management plan
85854	Review of patient at risk from coronary heart disease
95889	Assessing cardiovascular risk using SIGN score
95948	QRISK cardiovascular disease 10 year risk score
96142	Cong heart fail, hypertens, age, diab, stroke 2 risk score
96886	Cardiovascular disease risk assessment done
96899	Cardiovascular disease risk assessment indicated
97641	Cardiovascular disease risk assessment
98113	QRISK2 cardiovascular disease 10 year risk score
98120	Framingham 1991 cardiovascular disease 10 year risk score
98429	Cardiovascular disease high risk review
100937	CVD (cardiovascular disease) risk assessment by third party
101644	Consent given for cardiovascular health risk assessment
104175	Joint British Societies cardiovascular disease risk score
105223	At risk of cardiovascular disease
105901	High risk of cardiovascular disease

Amendments to original protocol (changes approved by ISAC in December 2015)

1) We focussed on comparing changes in the level and trend of statin initiation/cessation after the exposure period compared with before for our primary analysis, rather than estimating changes at the beginning and end of the exposure period, which would have been driven by the imprecisely estimated trend in the interim period (estimated using only 4 data points)

2) We used drugs prescribed for glaucoma as an exposure for the negative control analysis. This analysis was changed from our original negative control exposures of beta-blockers/ACE inhibitors as we believed there to be too large an overlap in the use of statins and ACE inhibitors/beta-blockers meaning that changes in prescribing of each could be related. Like statins, glaucoma drugs are prescribed to those at high risk of disease as a preventative measure and are typically intended to be continued for life after initiation, but they have the advantage (for our purposes) of a completely unrelated indication.

3) As well as adjusting for seasonality and autocorrelation, we scaled standard errors using the square root of the Pearson chi-squared based dispersion scalar to account for over dispersion in all main analyses.

4) We conducted a post-hoc cessation analysis which separated post-exposure time into two six month periods. This decision was made because the observed data points in the analyses for cessation in primary and secondary prevention suggested an initial change after the exposure period that later attenuated, and executing these post-hoc analyses allowed us to assess if levels of cessation returned to a level similar to pre-exposure after six months. These post-hoc analyses were a more accurate representation of the trends in cessation, and so were used to estimate public health impact.

5) Another tool we used to explore the initial change in cessation for primary and secondary prevention, which later attenuated, was to carry out post-hoc analyses investigating both a step change in level and a trend change simultaneously. This allowed us to determine a monthly rate at which cessation fell after the initial increase in level.

6) To investigate an observed decrease in the number of patients receiving a cardiovascular disease risk score of ≥20% following the exposure time period (the denominator for initiation analyses in primary prevention), we explored the proportion of patients with any recorded 10 year CVD risk score in the whole CPRD population each month within our study period. We then investigated whether patients were categorised as at very high (≥30%), high (20-30%%), intermediate (10-20%) or low (<10%) risk of CVD.

7) We conducted a secondary negative control analysis using an alternative exposure time period of 12 months earlier than our original exposure time period. This was because we had no reason to expect any changes in prescribing trends around this time.

8) As well as applying cardiovascular disease event rates and known statin efficacy estimates to the public health impact, we also corrected for the proportion of patients likely to have stopped statin therapy over 10 years regardless of the media controversy (estimated from historical CPRD data), and published post-cessation restart rates.
